# Using Machine Learning to Analyze Molecular Dynamics Simulations of Biomolecules

**DOI:** 10.1021/acs.jpcb.4c08824

**Published:** 2025-05-27

**Authors:** Alfie-Louise R. Brownless, Elisa Rheaume, Katie M. Kuo, Shina C. L. Kamerlin, James C. Gumbart

**Affiliations:** † Interdisciplinary Graduate Program in Quantitative Biosciences, Georgia Institute of Technology, Atlanta, Georgia 30332, United States; ‡ School of Chemistry and Biochemistry, Georgia Institute of Technology, Atlanta, Georgia 30332, United States; § School of Physics, Georgia Institute of Technology, Atlanta, Georgia 30332, United States

## Abstract

Machine learning (ML) techniques have become powerful tools in both industrial and academic settings. Their ability to facilitate analysis of complex data and generation of predictive insights is transforming how scientific problems are approached across a wide range of disciplines. In this tutorial, we present a cursory introduction to three widely used ML techniques—logistic regression, random forest, and multilayer perceptron—applied toward analyzing molecular dynamics (MD) trajectory data. We employ our chosen ML models to the study of the SARS-CoV-2 spike protein receptor binding domain interacting with the receptor ACE2. We develop a pipeline for processing MD simulation trajectory data and identifying residues that significantly impact the stability of the complex.

## Graphical abstract

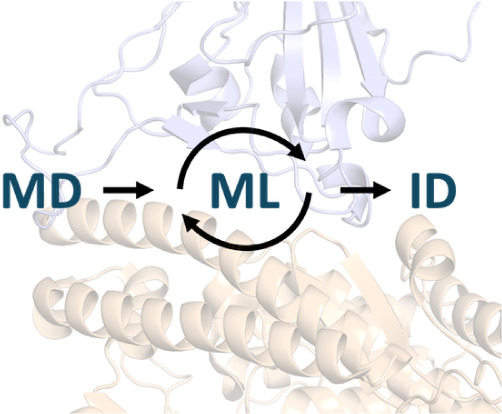

## Background

A novel coronavirus, severe acute respiratory syndrome coronavirus 2 (SARS-CoV-2), rapidly spread throughout the world since 2019.^1,2^ SARS-CoV-2 is less deadly but more transmissible than SARS-CoV, which appeared in late 2002.^1,2^ The membrane-enveloped coronavirus has a spherical shape with oblong protrusions called spike proteins littering the surface. To initiate infection, one spike protein’s ectodomain must contact its receptor, the human angiotensin-converting enzyme 2 (ACE2).^3–8^ The region of the spike protein that makes contact with the ACE2 receptor is called the receptor binding domain (RBD) and, intuitively, alteration of the structure of this domain impacts ACE2 binding and thus infectivity (Figure 1).^3–5,9^ Although a large portion of the SARS-CoV spike protein sequence is conserved in SARS-CoV-2, there are a substantial number of differing residues (∼50%) within the RBD that contribute to differences in binding affinity between the two variants,^4,7,8^ X-ray crystallography and cryo-electron microscopy experimental methods in combination with biophysical simulations allow for rigorous analysis of the differences in interactions among the two SARS variants at the atomistic level. Studies have shown that the SARS-CoV-2 RBD binds preferentially to the ACE2 receptor when compared to SARS-CoV, resulting in a more stable complex.^10–12^ Furthermore, particular mutations of the RBD that serve to stabilize RBD-ACE2 complex interactions will correspond to higher binding affinity^13^ and so understanding the stabilization of this structure due to changes in sequence is crucial in understanding changes in infectivity. The stability of the RBD alone can vary dramatically dependent on mutation, with some mutations exhibiting less stable structures and some, such as the Omicron N501Y mutation, increasing stability.^14^ MD simulations have been used to quantify these differences in interaction strength, which contribute to the infectivity of the virus.^9,12,15–17^ Fatouros et al. used computational methods to quantify ACE2 binding affinities despite lacking experimental structures.^18^ Additionally, Kumar et al. utilized computational methods to determine which mutations in the Delta and Omicron variants are responsible for their differences in binding.^14^ Furthermore, Jena et al. utilized MD simulations to understand how compounds catechin and curcumin can exhibit antiviral properties via differential binding to the RBD-ACE2 complex.^19^ A common theme surrounding these studies is the strategic use of computational methods to analyze complex, multidimensional data and rapidly address current public health crises. Here, we focus on a previous study by Pavlova et al.^15^ in which MD simulations were utilized in order to determine which residues are most important for distinguishing between the SARS-CoV and SARS-CoV-2 variants. Since analyzing each residue’s contribution individually is practically infeasible, they utilized three different ML algorithms to extract which residues contribute the most to the difference in binding affinity between SARS-CoV and SARS-CoV-2 and, thus, the increase in infectivity.

**Figure 1:**
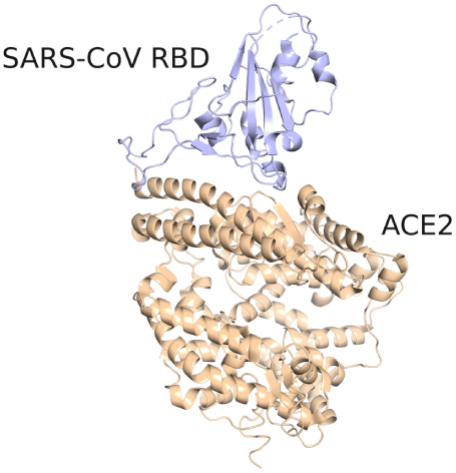
Visualization of the SARS-CoV RBD in complex with the extracellular domain of the ACE2 receptor. Molecular images rendered with PyMOL^20^ and VMD.^21^

ML methods provide great utility when parsing through very large data sets. In particular, they prove incredibly useful when drawing conclusions from methodologies such as MD simulations^22^ which result in gigabytes (or more) of atomistic coordinate data.^23^ Numerous groups have taken advantage of this fact and utilized various ML methods in order to analyze complex trajectory data and create useful tools to aid others in this process.^22–34^ In particular, we focus on the utilization of ML methods in order to perform advanced and rigorous structural classifications using MD data. Following the prior study by Pavlova et al.,^15^ we provide a python tutorial showcasing the utility of three different ML models to compare how the SARS-CoV and SARS-CoV-2 RBDs differentially bind to the ACE2 receptor.

## Theory

We utilize three different ML methods in order to adequately determine what structural factors allow for the increased binding affinity of the SARS-CoV-2 RBD to the ACE2 receptor. Generally, ML models are useful tools in determining patterns associated with very large collections of data. In this case, we will apply an ML method in order to more easily and rigorously quantify the differences in dynamics between the SARS-CoV and SARS-CoV-2 RBDs interacting with ACE2 that would not be clear from visual observation alone. More generally, input vectors, or “features”, are mapped to output values or “classifications”, and a “training” process occurs whereby weights of input values are adjusted in order to minimize error in outputted classifications. We utilize supervised learning algorithms where we provide categorical classifications of training data up front. In this case, the classifications correspond to either the SARS-CoV or SARS-CoV-2 RBD. Our process of model training involves splitting our data into testing and training components (testing to determine weights and training to adjudicate the accuracy of the results), optimizing parameters based on minimizing error in training data, and then evaluating performance (Figure 2).

**Figure 2:**
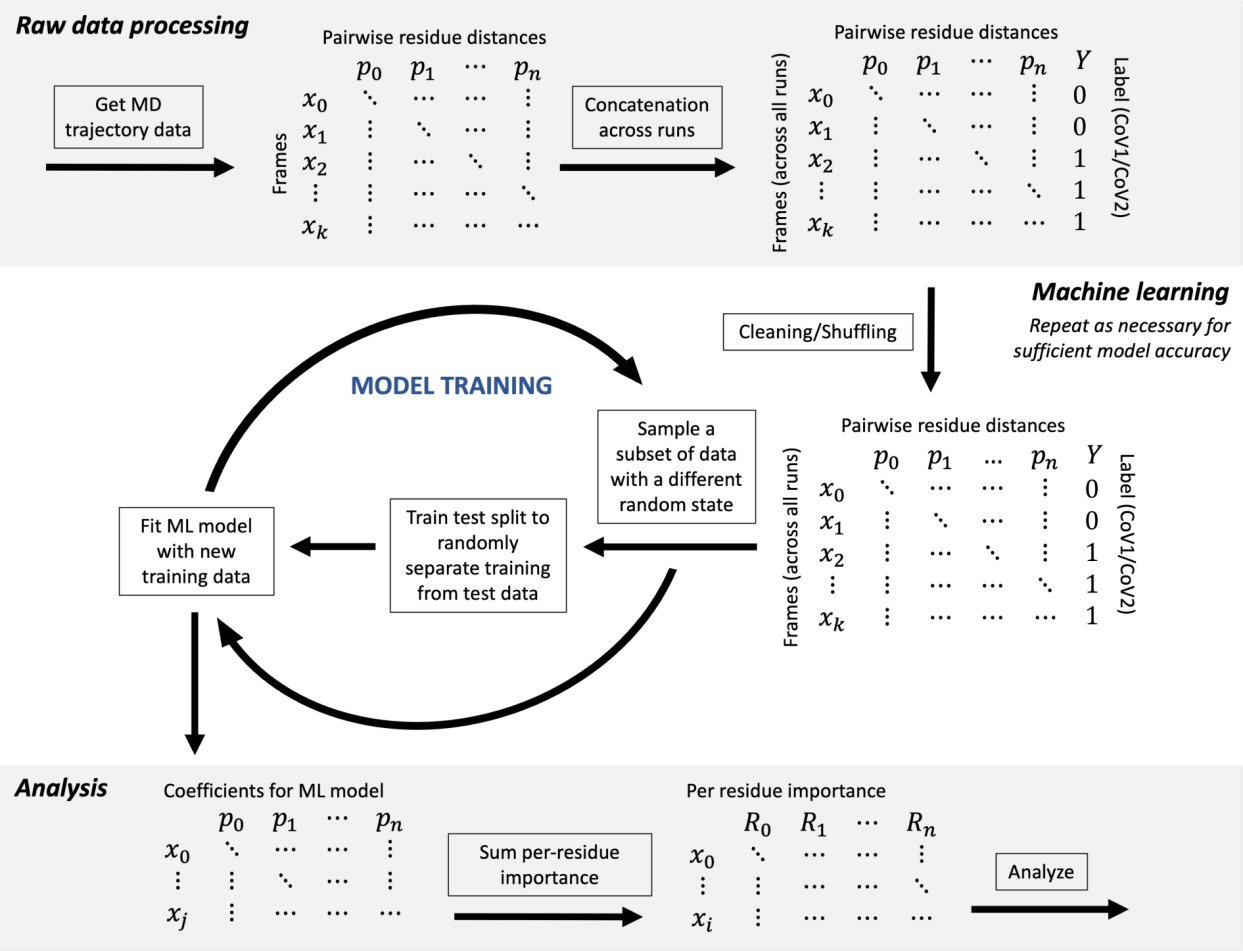
Schematic visualizing the pathway of data processing and analysis covered within this tutorial.

MD simulations were previously conducted with NAMD,^35^ sampling both the SARS-CoV and SARS-CoV-2 RBDs,^15^ and supervised ML algorithms were utilized to output which residues are most important to differential binding affinities. Here, we provide explicit instructions and advice in regards to the implementation of these supervised learning ML methods toward drawing conclusions from MD trajectories. More specifically, this tutorial focuses on the implementation of logistic regression, random forest, and multilayer perceptron methods in order to determine residue importance for distinguishing between RBD-ACE2 complexes. While not exhaustive, these three methods represent commonly used approaches for classification problems. We provide brief theory on the fundamentals of each of these three methods in the following sections.

### Logistic Regression

To describe logistic regression models, we first begin by introducing linear regression models. Linear regression is a technique whereby a response variable is estimated by a linear function of a corresponding explanatory variable;^36^ in other words, if we predict that a classification *y* is linearly related to our descriptor variables *x*_0_, *x*_1_,...,*x*_*N*_, then we can use this known relationship to determine any classification *y* based upon any arbitrary series of inputs. Linear regression models, therefore, can output an estimation of a given value, *y*, based on an input, *X*, assuming a linear relationship between input and output variables.^37^ In this case, we assume that X = *x*_1_, *x*_2_,...,*x*_*N*_ can adequately describe *y* via the following relationship

$$y=\beta_{0}+\beta_{1}x_{1}+\beta_{2}x_{2}+...\beta_{N}x_{N}+\varepsilon$$

Importantly, a linear regression model produces a continuous response variable and thus is not useful for our purposes (since we only need a binary classification). However, we can apply a generalized linear model (GLM), which is a type of model utilizing a fundamental logic similar to linear regression models, but expanded to include more general categorical target variables.^37^ This is done by constructing each linear term β_*i*_ *x*_*i*_ from a more complex functional form, typically an exponential.^37^ More specifically, we will be implementing a logistic regression model, which is an example of a simple GLM.

Here, let us classify distances associated with the SARS-CoV RBD as “0” and with the SARS-CoV-2 RBD as “1”. For each input vector (from 1,...*i*,...,*N*), we can define a probability that our input vector corresponds to the SARS-CoV-2 RBD (*y*_*i*_ = 1) as

$$\begin{array}{l} \pi_{i}=P\left\{y_{i}=1\right\}\;\mathrm{such}\;\mathrm{that}\\ E\left(y_{i}\right)=\pi_{i} \end{array}$$

In order to construct a linear combination of values with π_*i*_ as inputs, we require a final transformation function to ensure that our outputs will always smoothly produce a number between 0 and 1. Therefore, we finally define the following

$$\log\left(\frac{\pi_{i}}{1-\pi_{i}}\right)=\beta_{0}+\beta_{1}x_{1}+\beta_{2}x_{2}+...\beta_{N}x_{N}$$

which also takes the forms

$$\pi_{i}=\frac{1}{1+e^{-(\beta_{0}+\beta_{1}x_{1}+\beta_{2}x_{2}+...\beta_{N}x_{N})}}$$

and

$$\pi_{i}=\frac{e^{\beta_{0}+\beta_{1}x_{1}+\beta_{2}x_{2}+...\beta_{N}x_{N}}}{1+e^{\beta_{0}+\beta_{1}x_{1}+\beta_{2}x_{2}+...\beta_{N}x_{N}}}$$

Here, we will train our model to determine which weights (β_0_,···,β_*N*_) are optimal for accurately predicting a particular RBD variant given a series of input distances. We will then use these weights to help determine which residues are most important in differentiating between SARS-CoV and SARS-CoV-2 RBDs.

### Random Forest

Decision tree classifiers are simple models where “decisions” are made sequentially in order to most optimally divide a given set of data into individual categories (classifications). Random forest classifiers are expansions on decision tree classifiers, whereby each “tree” is generated via the classification and regression tree (CART) algorithm.^38^ The CART algorithm allows for the implementation of a recursive division process with the goal to provide the least number of binary divisions required to adequately separate two (or more) target conditions.^38^ Put more simply, a random forest model consists of a combination of decision trees, where each tree can make an individual prediction (in this case, predicting whether the given residue–residue pairing of interest belongs to SARS-CoV or SARS-CoV-2), and the combination of predictions across the multiple decision trees generates a more accurate answer.

Additionally, instead of generating one singular random forest prediction, bootstrap aggregating (bagging) is often used to reduce variance in outputted predictions.^38^ Bagging is an ensemble method where subsamples of data are randomly pulled from the original data set and corresponding predictions are generated. Once all predictions have been constructed, they are combined to obtain the final random forest model. This procedure is appreciated since, when applied, there will consistently remain unused “out of bag” (OOB) data consisting of approximately 37% of given data.^38^ This OOB data can then be used as effective testing data to validate a random forest model. Similarly to our logistic regression model, we can use the weights associated with each feature (and how each feature impacts the trained decision tree) in order to pinpoint which residues contribute more to the differences in binding between the two virus’s RBDs.

### Multilayer Perceptron

A multilayer perceptron (MLP) is a neural network containing input nodes, hidden layers, and output layers connected by corresponding weights.^39^ Simply put, a neural network consists of layers of nodes, whereby each node processes information, which is then propagated to nodes in the next layer. The combination of values from the final layer are used in order to determine the prediction that the network outputs.

Neural networks are incredibly flexible models due to the ability to take in information from a series of inputs and combine the results in a variety of different ways, implementing connections among nodes that propagate across multiple hidden layers, allowing for an inherently nonlinear response to be produced. More specifically, each neuron is partially defined by a summation function, typically of the form $S_{j}=\overset{n}{\underset{i=1}{\sum }}w_{ij}I_{j}+\beta_{j}$ , which combines all information obtained from incoming neuronal connections.^39^ Additionally, each neuron will then take in the summed information and produce an output based upon an activation function, which is typically a smooth sigmoidal function.^39^

## Prerequisites

To gain the most from this tutorial, prior experience in Python is suggested for users. We recommend the user begin with a Python environment containing these packages:

- scikit-learn 1.2.2
- pandas 1.5.3
- Numpy 1.23.5
- Jupyter Notebook 6.5.2
- Matplotlib 3.6.2

## Exercises

In this tutorial, we provide two data sets, each containing pairwise distances of nearby residues between the spike-protein RBD and the ACE2 receptor for both SARS-CoV and SARS-CoV-2 (Figure 3); in addition to processed data sets, original simulation trajectories are available online at Zenodo (DOI: 10.5281/zenodo.15376189). We also provide residue–residue pairing information corresponding to each index within the distance matrices. The data were collected from two independent runs, each consisting of 2 μs MD simulations, initialized for both SARS-CoV and SARS-CoV-2 RBDs in complex with the ACE2 receptor. In order to reduce computational burden, each data set contains distances collected across only 1000 frames evenly spaced across the corresponding trajectories. We note that increasing the number of processed frames can increase the accuracy of calculated residue importances.

**Figure 3:**
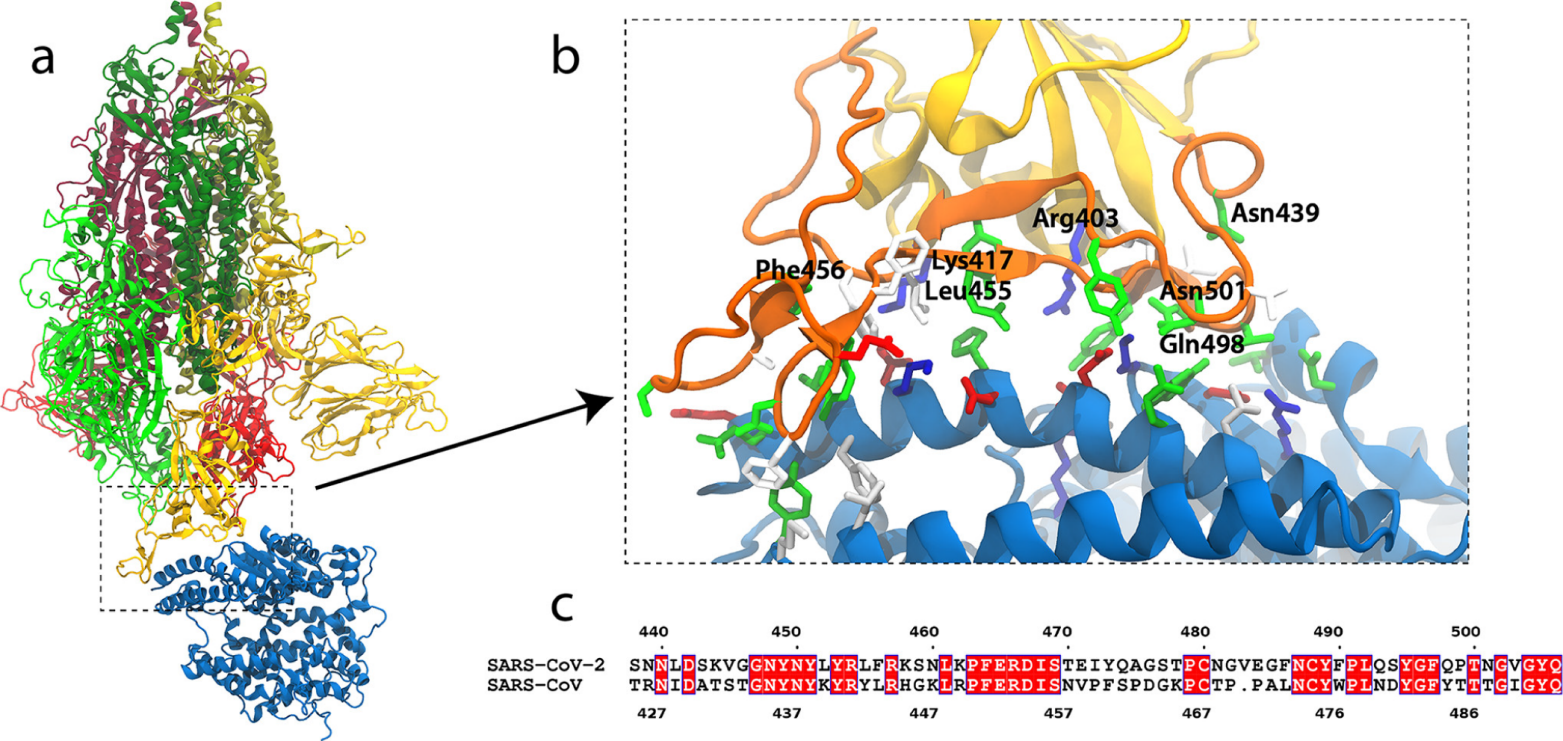
(a) SARS-CoV-2 spike protein (red, green, and yellow) bound to the ACE2 receptor (blue). (b) Closer view of the RBD-ACE2 interaction scaffold. Interacting residues are colored by residue type (red/blue: charged, green: polar, white: hydrophobic). (c) Sequence alignment of the SARS-CoV and SARS-CoV-2 receptor binding motif (RBM) with identical residues highlighted in red. The figure was reproduced from Figure 1 in ref 15. Copyright 2021 American Chemical Society.

Our data sets require a nontrivial amount of preprocessing in order to effectively train our ML models. This will function as the first exercise.

### Exercise: Data Preprocessing

We begin by creating a pandas dataframe containing all of the pairwise distances calculated across simulation time for two independent runs of SARS-CoV and SARS-CoV-2.
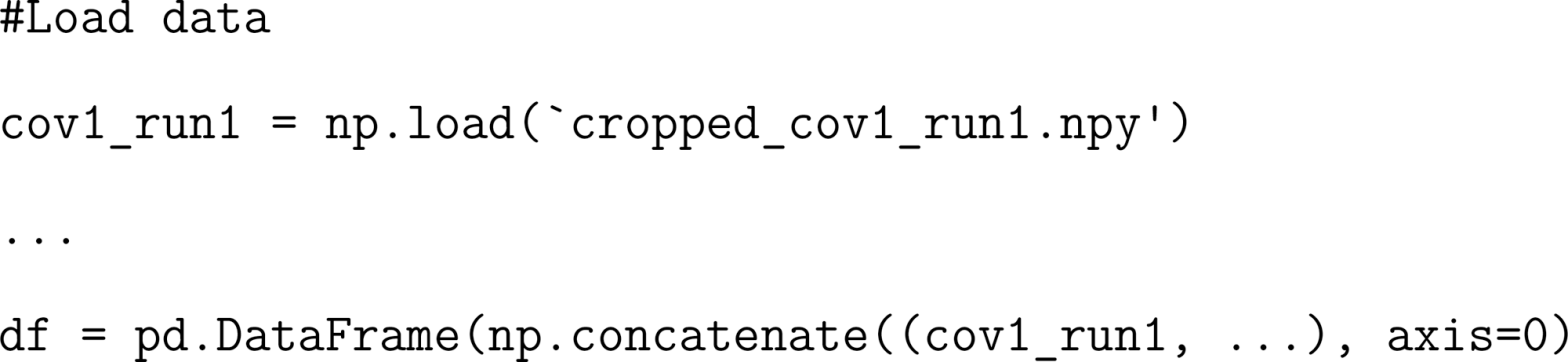


To increase the accuracy of model predictions, it is common practice in ML analytics to implement a standard scalar in the data pipeline (applied to training and test data before being fed into the ML algorithm), which removes the mean and scales to standardize variance.

In our case, as residues that are closest in physical space are likely to interact and therefore have a greater impact on the dynamics of the binding interface, it is reasonable to define co-residue importance by an inverse-distance relationship. Therefore, after storing our data, we take the inverse of each data point and scale our entire dataframe, obtaining a coarse measurement which will later be used to define residue “importance” for each residue pairing.
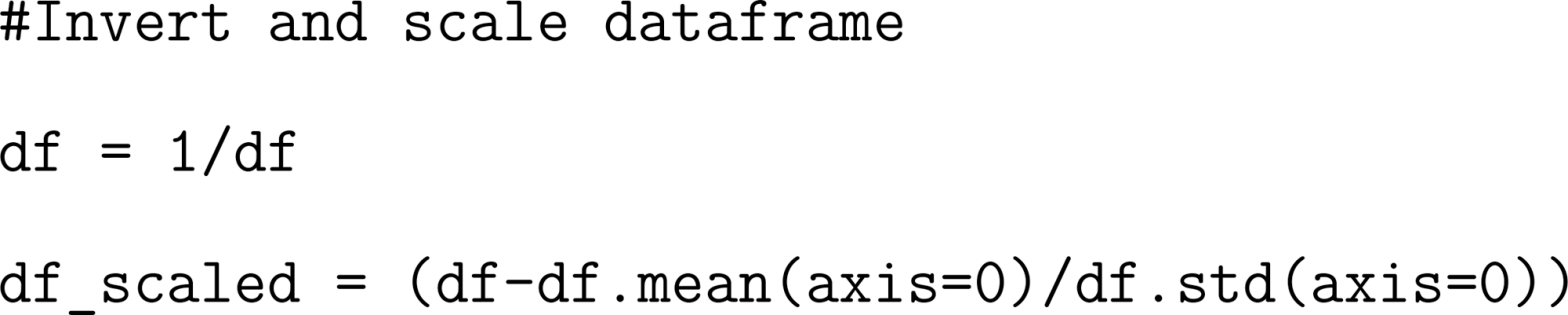


Finally, in order to conduct our supervised ML, we need to add a target variable classification to each of our frames that corresponds to the given RBD variant.
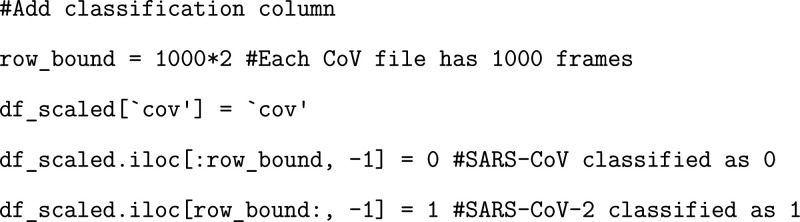


When we start to train our models, we want to remove any features that share a very high correlation, since correlated features are redundant and greatly increase the computational burden of our analysis. We generate a correlation matrix corresponding to the original dataframe and plot the initial correlation matrix to visually observe the areas of high correlation (Figure 4).
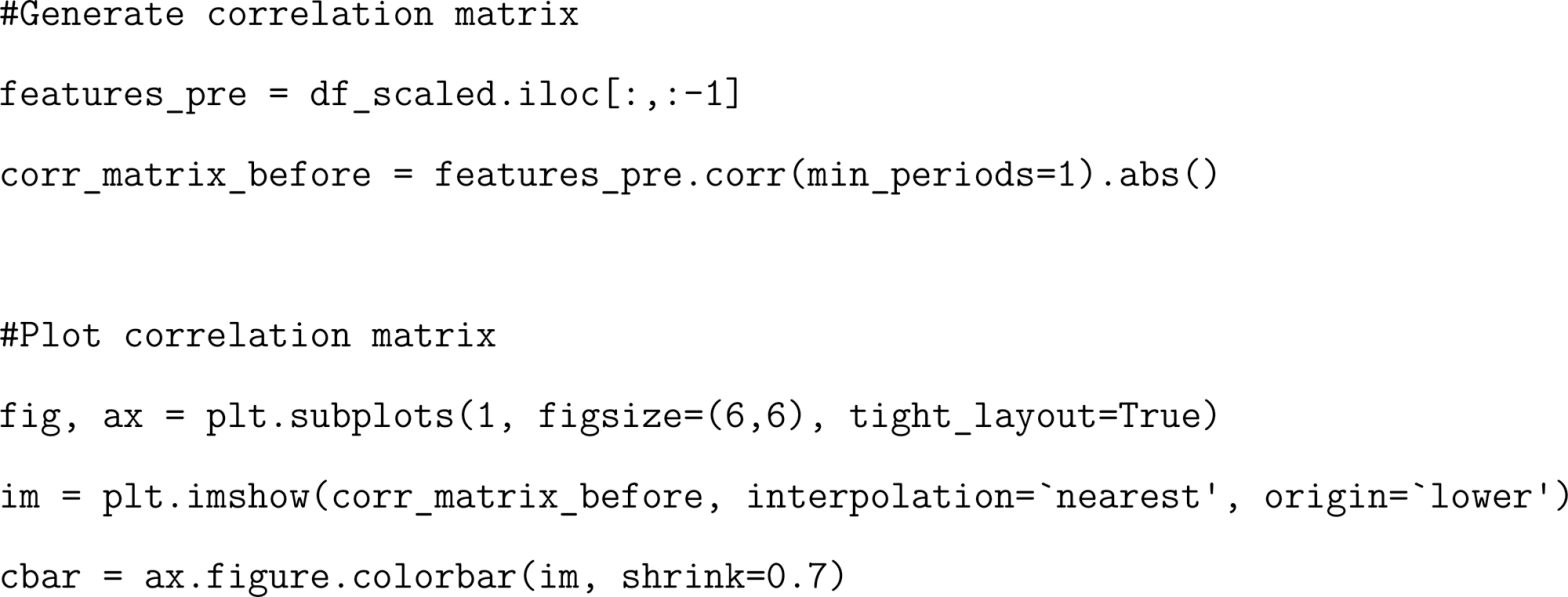


**Figure 4:**
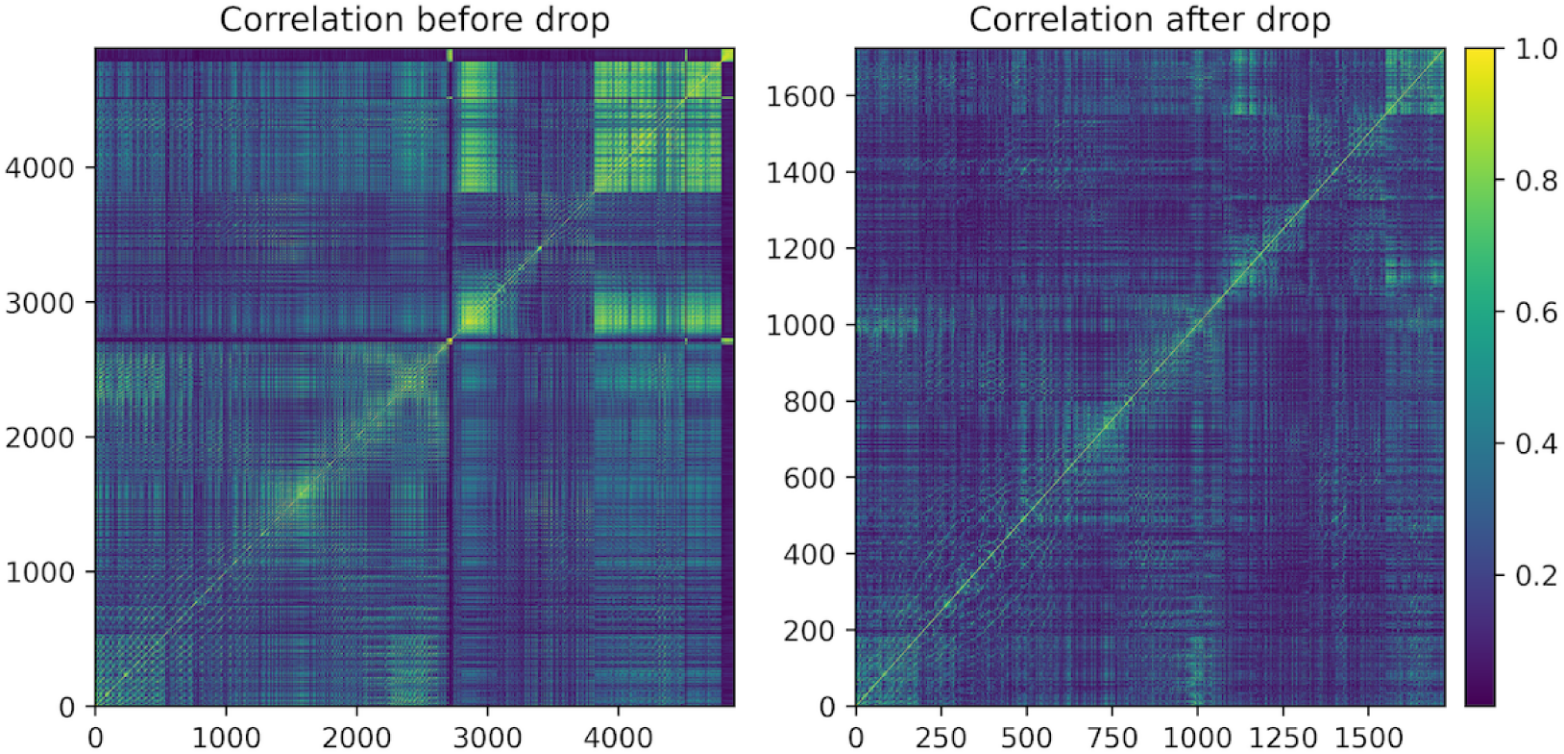
Correlation matrices before and after dropping highly correlated data.

We then shuffle the correlation matrix and drop any features that fall above the correlation threshold.
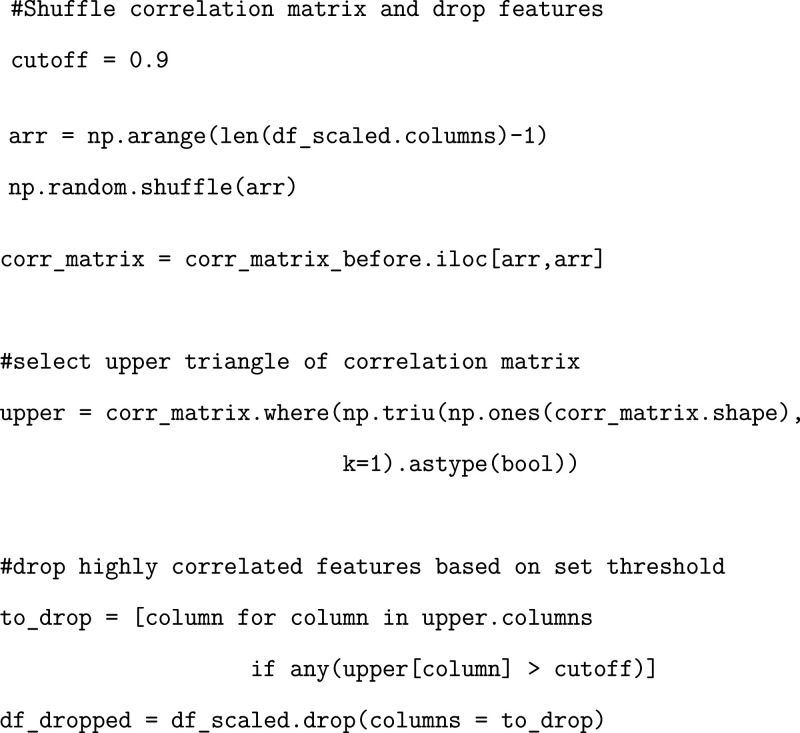


We plot the resulting correlation matrix to visually inspect the results and to showcase the amount of data that has been removed from the dropping process (Figure 4). Importantly, multiple runs should be conducted of the ML training where each run corresponds to a different reshuffling of the correlation matrix. This is important as it will allow for different combinations of correlated residue pairings to be removed, allowing for a much more stable end result. We provide exercises for the user to conduct only one run, but we strongly recommend implementing multiple correlation reshuffling in practice.

Finally, we initialize an instance of logistic regression, random forest, and MLP models utilizing a series of defined parameters. We note that the ML parameters chosen for this tutorial were previously optimized by performing a sweep across numerous value ranges and choosing those that gave the best performances. We leave the user with the additional exercise of determining optimal parameters choices if they so desire.
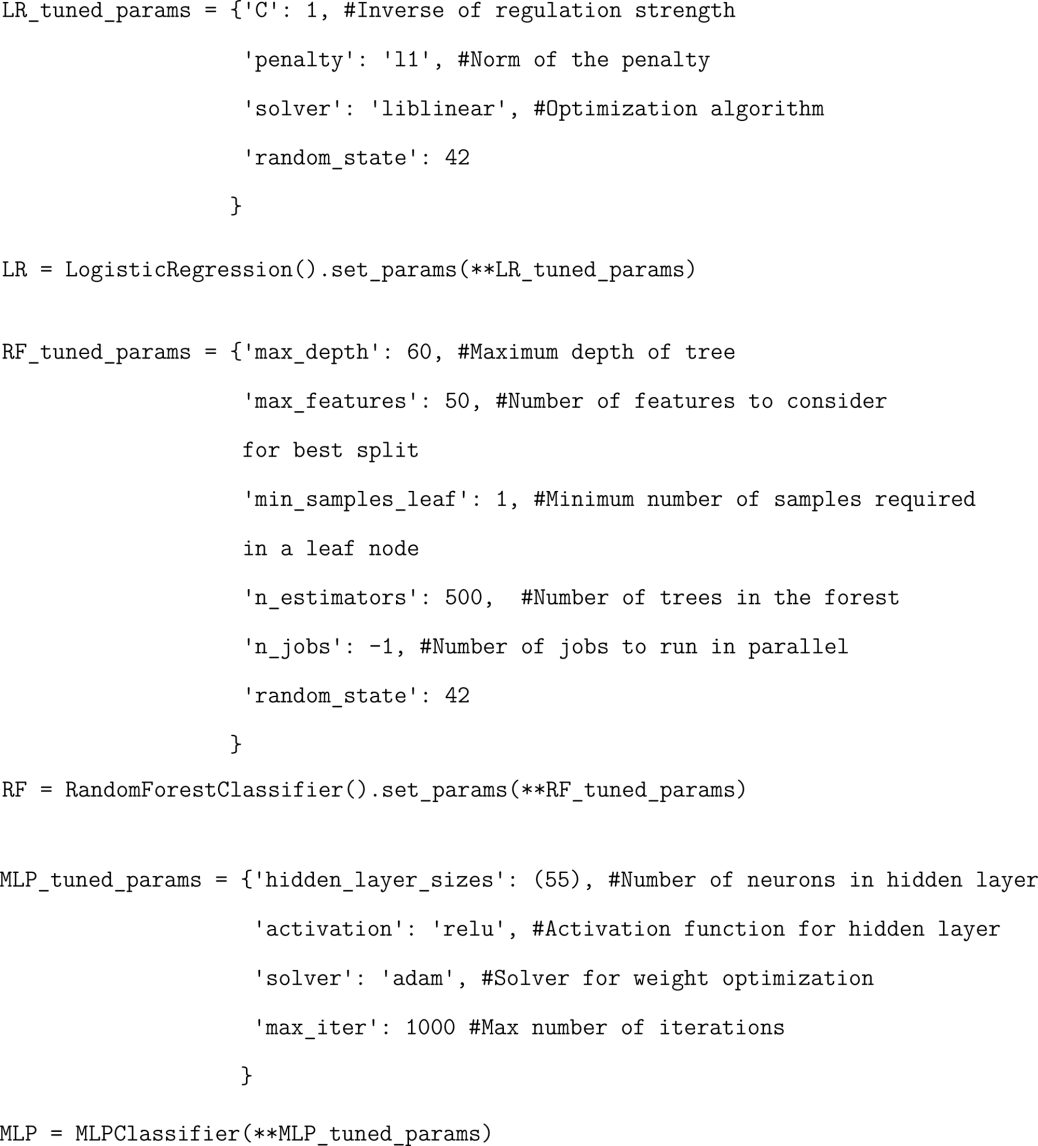


We recommend generating separate blank dataframes corresponding to the RBD and the ACE2 receptor residues that will be used to store summed residue importances.
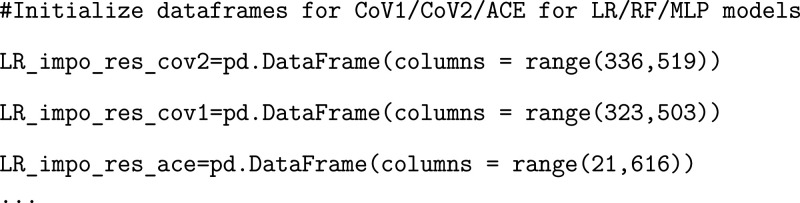


### Exercise: Calculating Residue Importance

Our dataframe contains information regarding the inverse distances associated with each residue pairing. We will then train our ML model to determine which residue pairs are most important in differentiating between SARS-CoV and SARS-CoV-2. These importances will be assigned to each residue pair and calculated from the coefficients of our trained LR model, the feature importances of our RF model, and relevance from a layer-wise relevance propagation method (explained in more detail below) for our MLP model. In this way, we treat each residue pair as a feature and then can quantify how “important” each individual residue is based on the importances of the corresponding residue pairs. In order to do this, we provide an exercise to generate a simple function that sums all of the inverse distances associated with each residue across the entire dataframe. We also provide a different method of determining per-residue importance factors, which could potentially (but not necessarily) alter the conclusions from each model. This function will be utilized in later components of the tutorial.
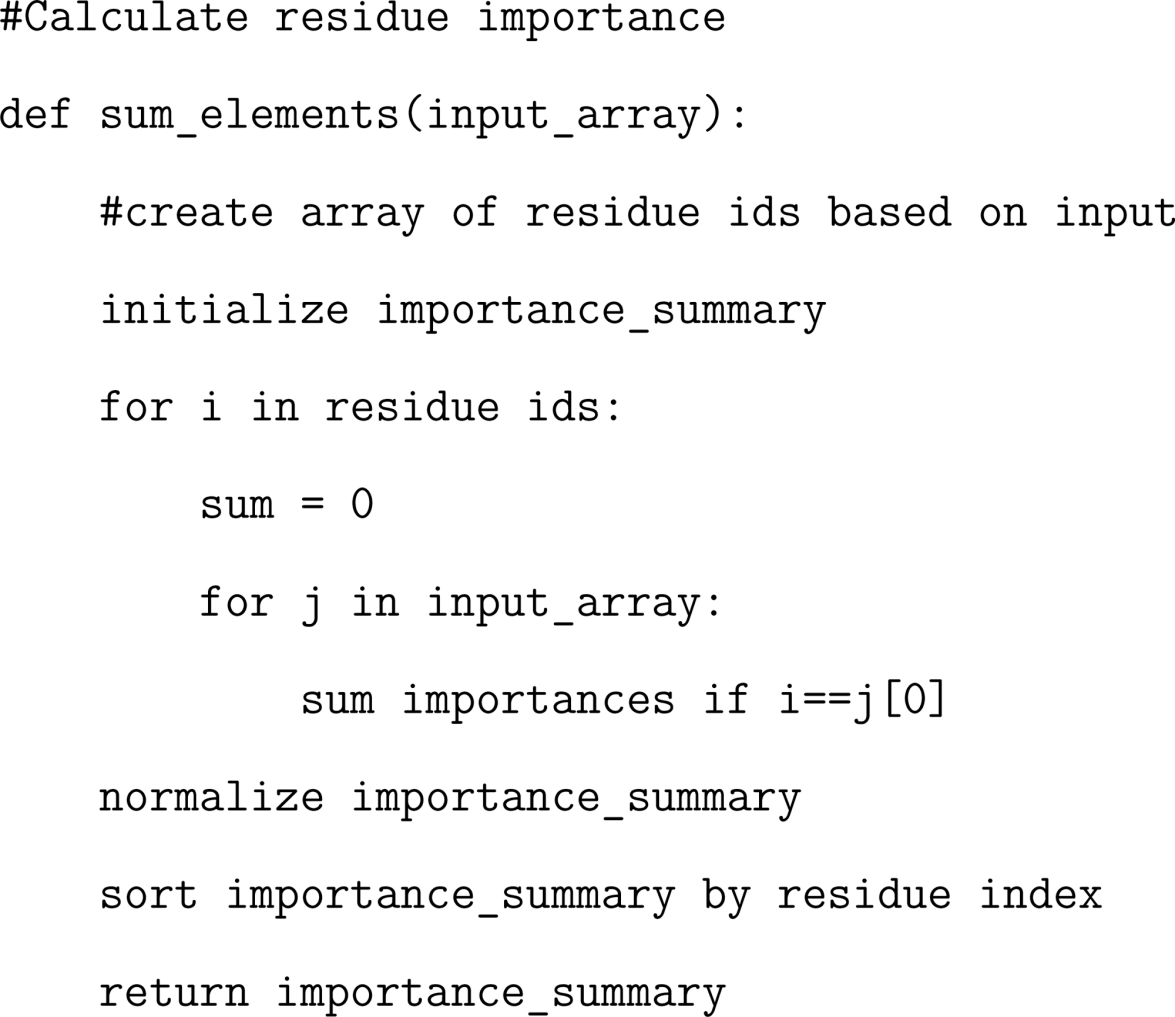


### Exercise: Developing a Trained Logistic Regression Model

Generally, it is standard practice to split any training data into “training” and “testing” inputs such that the performance of the model can be easily evaluated after sufficient training.^40^ The percentage of data used for the training set can vary, but we suggest using an 80/20 training/testing split. This exercise splits the data into a training and testing component and fits a logistic regression model to the training data. We point out that one should loop through multiple sets of random states to increase convergence.
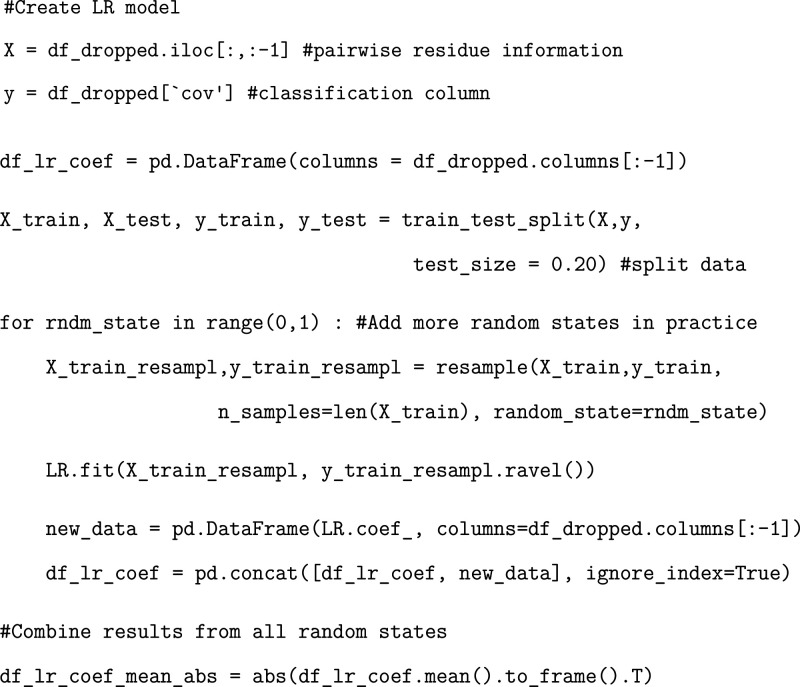


### Exercise: Evaluating Logistic Regression Model Performance

Evaluating the accuracy of a generated model is an important and useful procedure. We recommend implementing a variety of tests in order to evaluate each ML model. Once the model reaches sufficient accuracy (up to 100% as targeted here), we can move forward with our study.

We note that when training our models, we were able to achieve a sufficiently high accuracy in all evaluation metrics. However, there are cases in which that is not possible to achieve. In these situations, it may be necessary to prioritize one score over another. “Accuracy” is defined as the ratio of all true positives and true negatives to the total number of samples.^41^ This metric gives a simple indication as to how many correct classifications were made, but it can easily be skewed by uneven sizes in data sets.^41^ For our purposes, this is not a problem because we ensured that the number of trajectory frames corresponding to SARS-CoV and SARS-CoV-2 were equivalent. “Recall” describes the ratio of true positive classifications to all samples which should have been classified as positive.^41^ “Precision” is defined by the number of correctly assigned samples for a particular category (positive or negative) divided by the total number of samples in that category.^41^ This metric is very useful if incorrectly classified samples within a particular class (positive or negative) are more detrimental to the output of the model. The F1 score is the harmonic mean between precision and recall, and so it is a convenient metric to use if extreme values of precision or recall need to be taken into consideration to properly evaluate a model of interest.^41^ Finally, the “ROC_AUC” (receiver operating characteristic curve/area under the curve) metric takes into account how effectively a model can distinguish between both positive and negative cases^42,43^ which is useful in our case as we care equally about both the SARS-CoV and SARS-CoV-2 classifications.
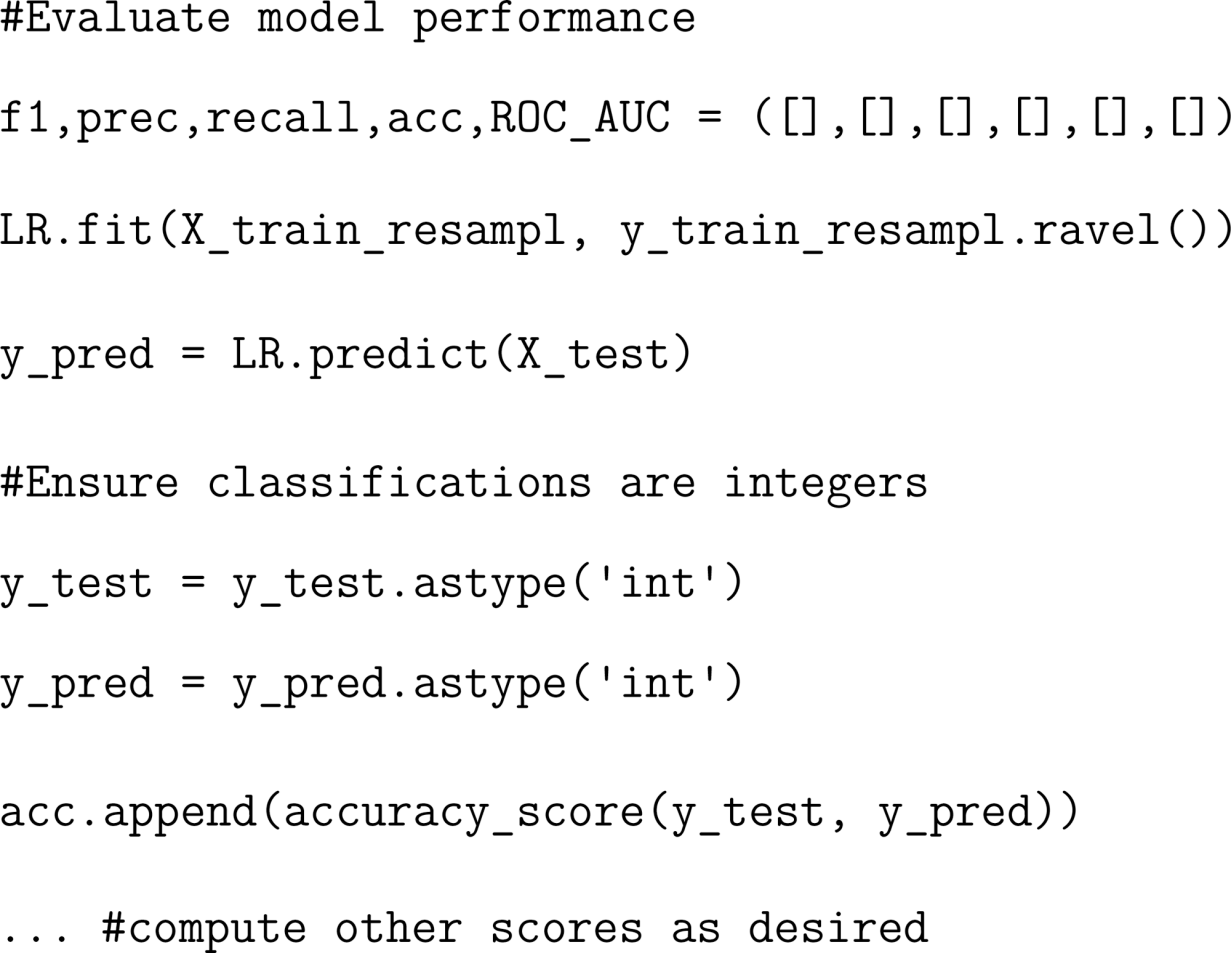


Plotting any of the performance metrics will show that our model has been well parametrized. We encourage the reader to adjust initialized parameters to see how sensitive the models are to changes in parametrization.

### Exercise: Determining Logistic Regression Per-Residue Importance

Here, we sort the pairwise residue importances by CoV, CoV-2, and ACE before adding these values to our summary dataframes.
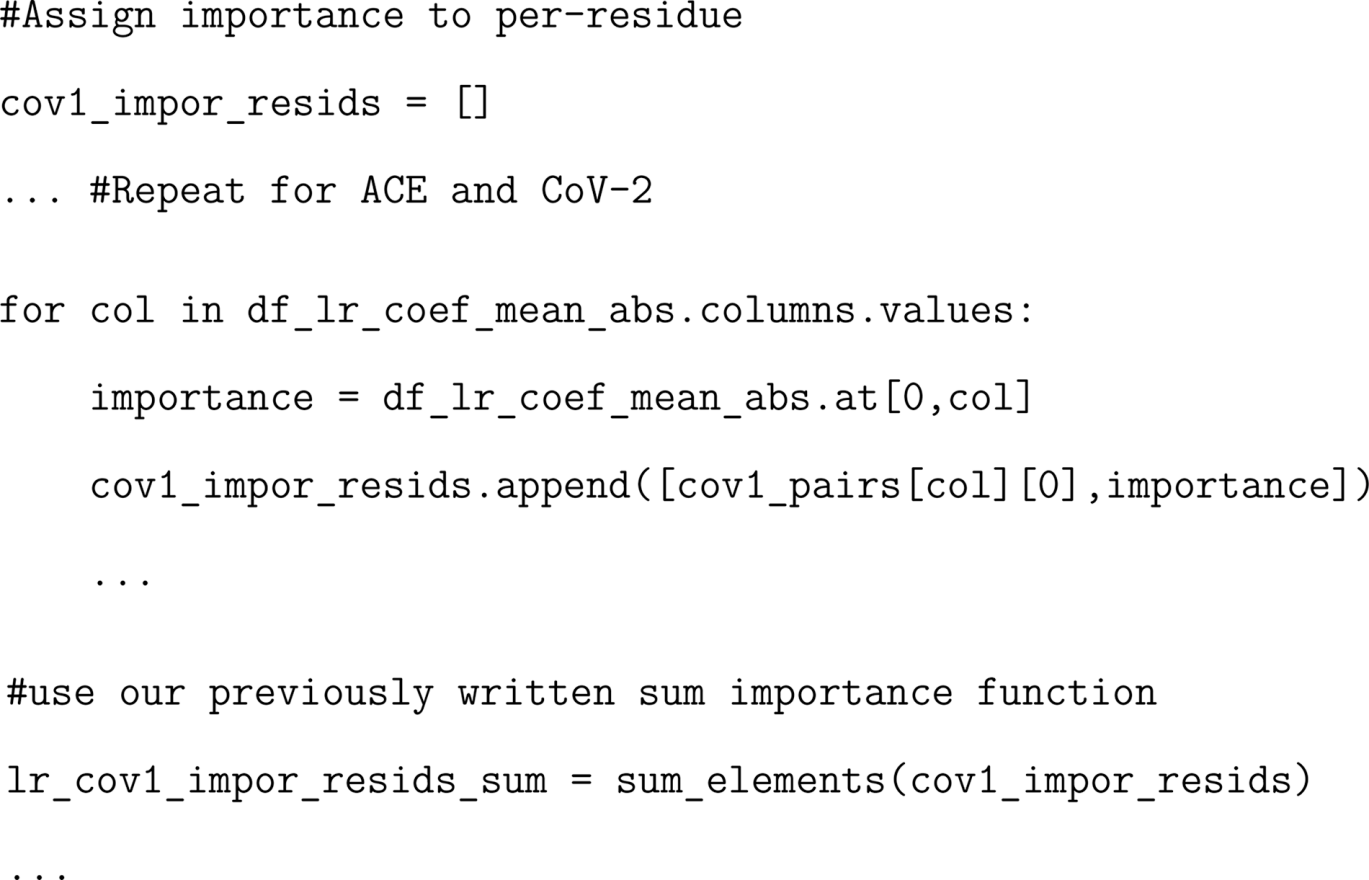


### Exercise: Developing and Evaluating a Trained Random Forest Model

We provide an exercise identical to the one above but utilizing the random forest model instead.
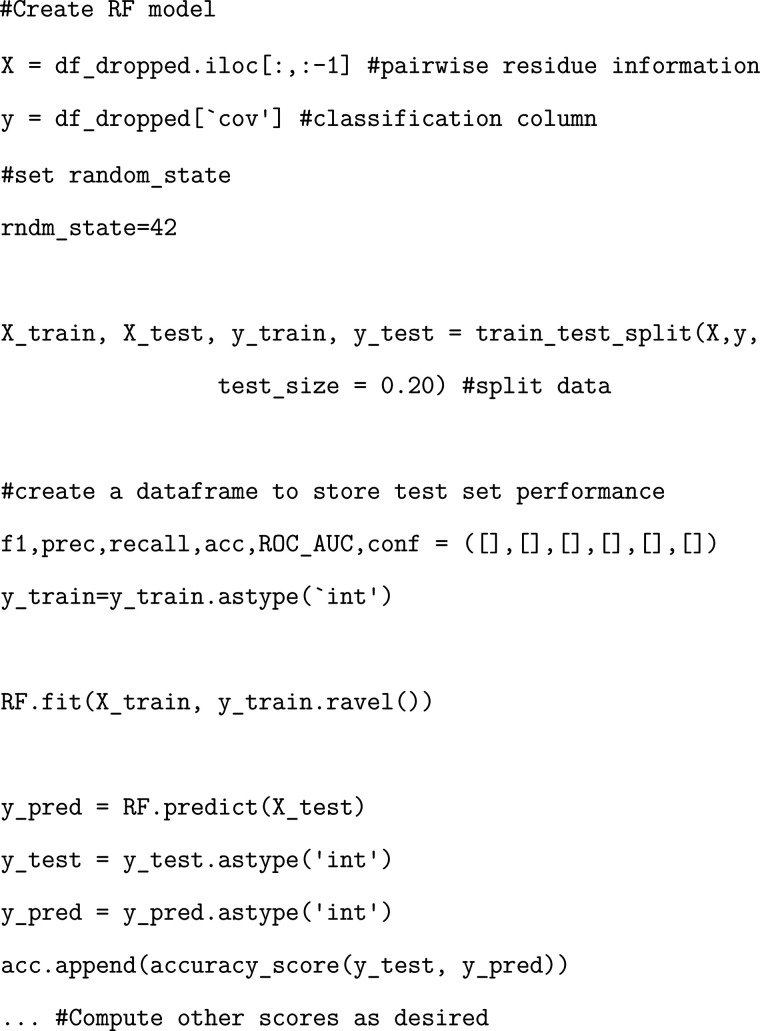


### Exercise: Determining Random Forest Per-Residue Importance

As before, we will extract the importance of each residue as determined by the Random Forest model.
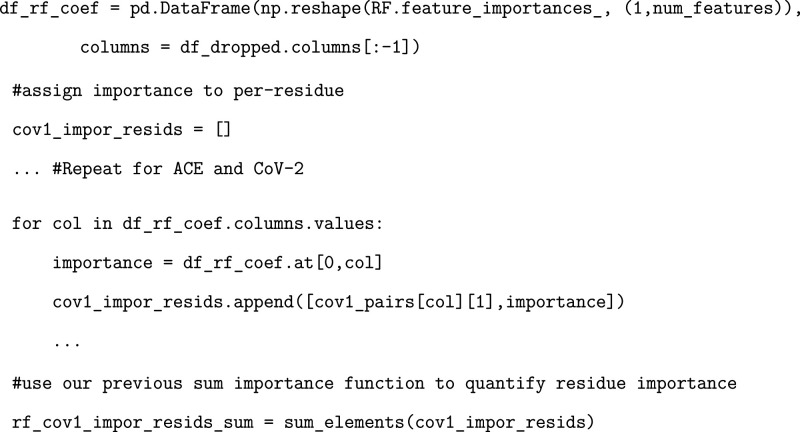


### Exercise: Developing a Trained Multilayer Perceptron Model

Finally, we showcase the utilization of a neural network MLP model. Training this model follows a very similar protocol to that of the other two models but with some key differences.

First, we transform our classification array using one-hot encoding. In order to increase the effectiveness of our MLP model, utilizing one-hot encoding is a common practice that creates a binary representation associated with each classification.^44^ We will transform our data in the following manner.
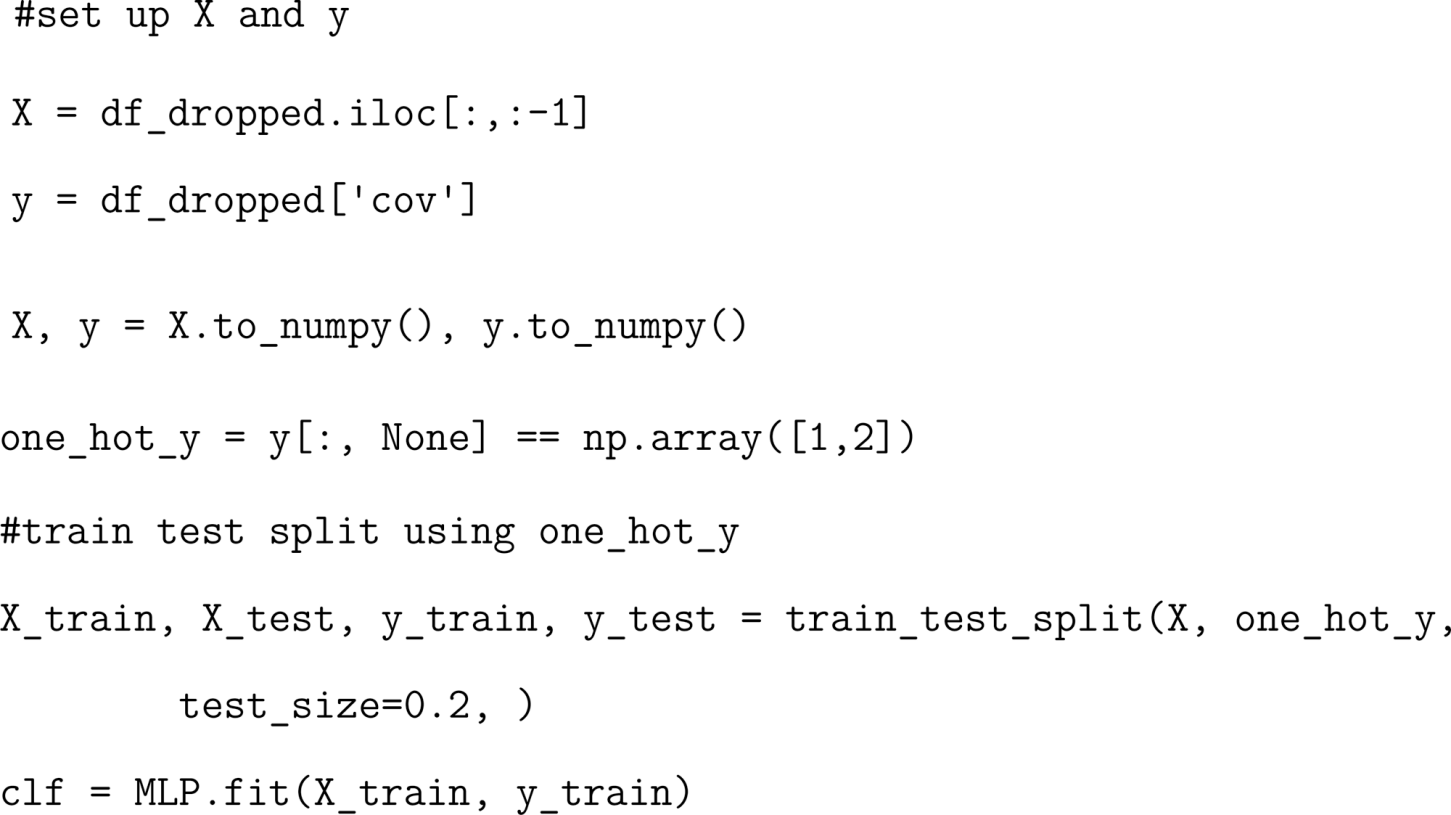


Once our model is trained, we cannot directly access residue pair importances like we were able to with our logistic regression and random forest models. Instead, to quantify the importances of each of our residue pairs, we need to utilize layer-wise relevance propagation, a technique to determine feature importance based on back-propagation of neural signals across layers of the network.^45^ With these differences in mind, we can proceed with training our MLP model.
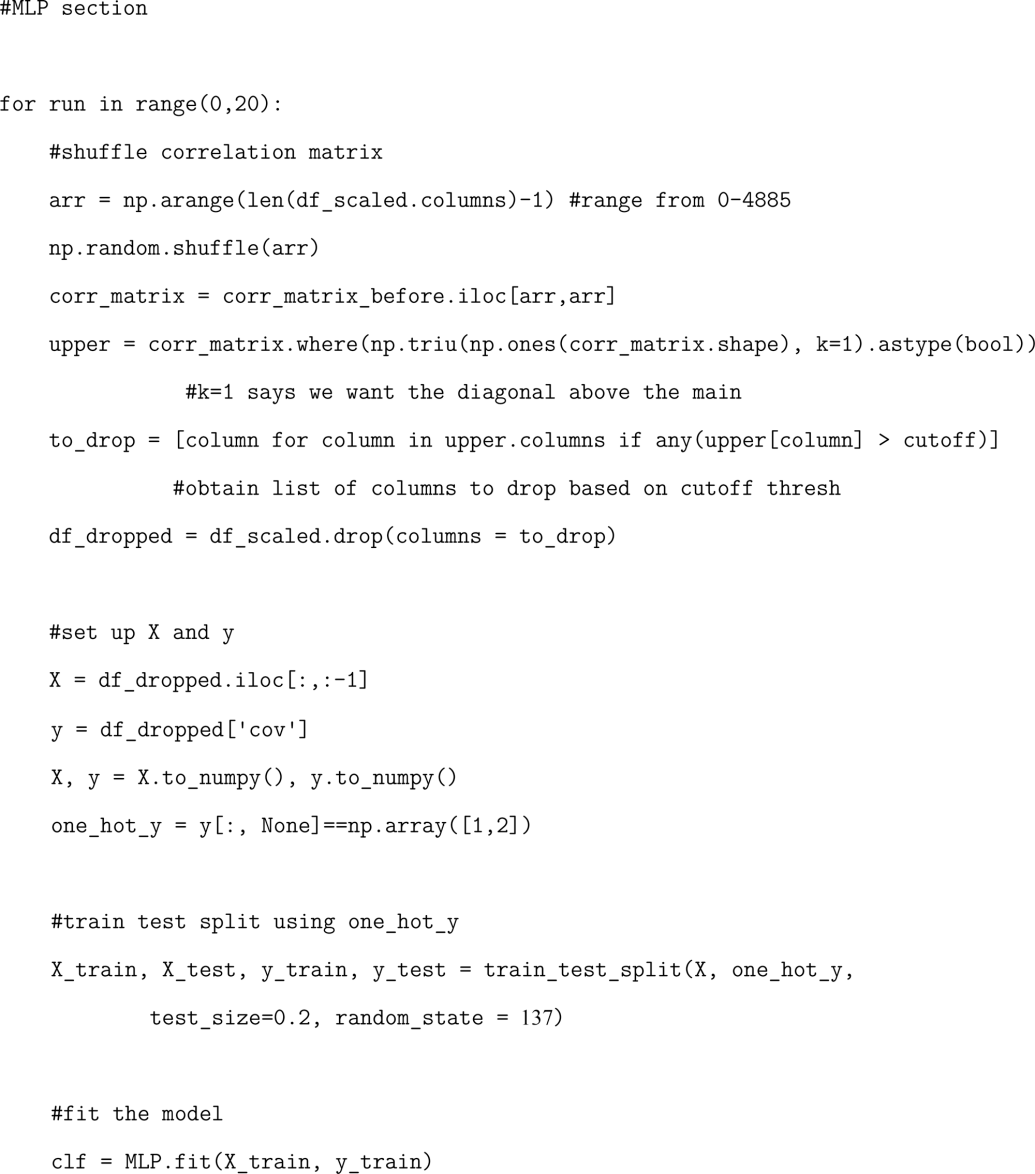


### Exercise: Evaluating MLP Model Performance

We will evaluate the accuracy of our MLP model in the same manner as we did for our logistic regression and random forest models.
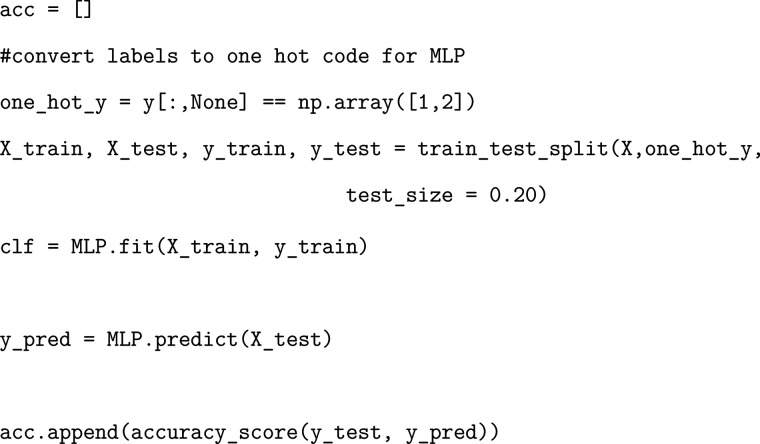


Once again, plotting the resulting accuracies will demonstrate the validity of our chosen initialization parameters.

### Exercise: Determining MLP Per-Residue Importance

Using layer-wise relevance propagation, we will obtain our residue importances calculated from the MLP model and add them to the corresponding dataframes.
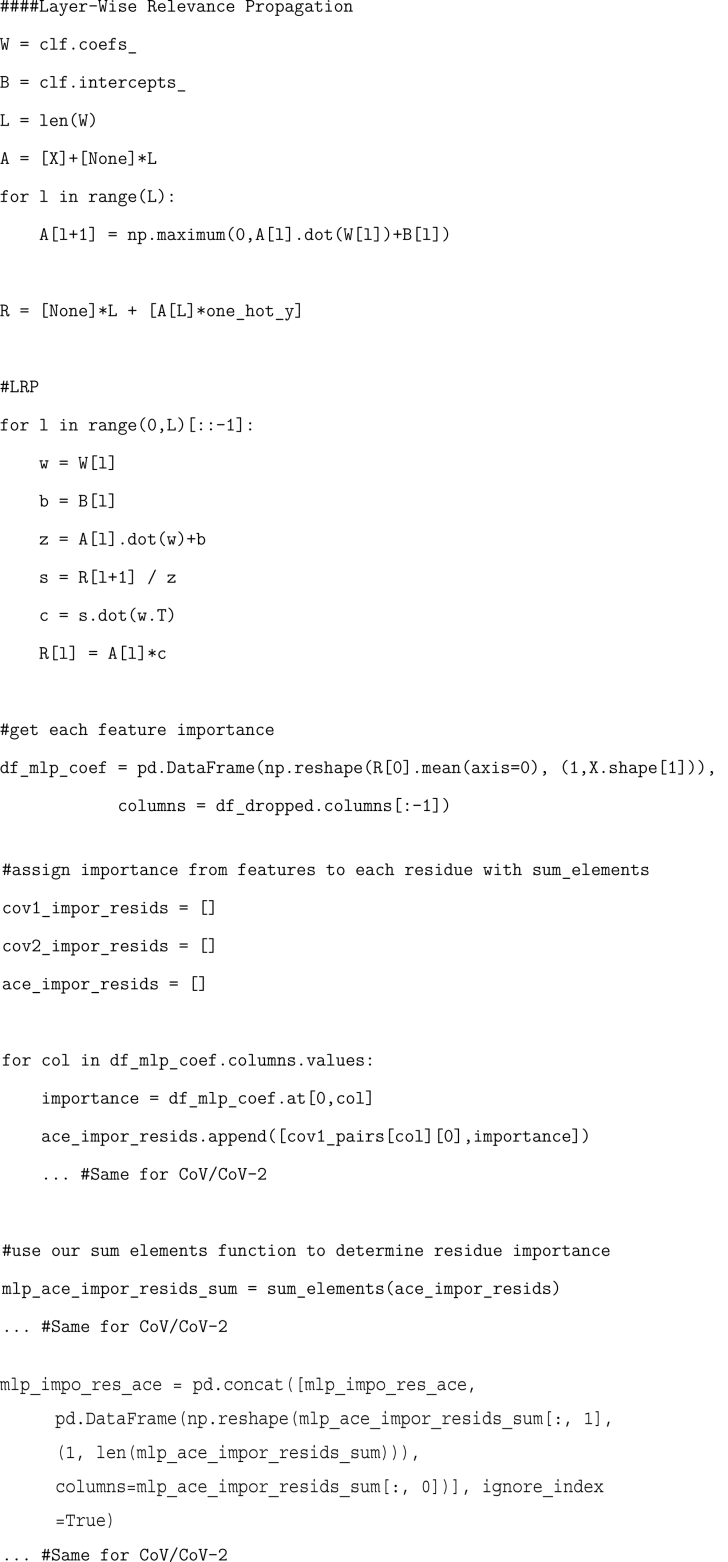


### Exercise: Plotting and Interpreting Results

Finally, we plot and interpret which residues are most important to differentiating the binding between SARS-CoV and SARS-CoV-2 RBDs to ACE2 as determined via our three ML models (Figure 5). Once highly important residues are determined, their importances can be projected onto the protein structure to better visualize locations of greatest importance.
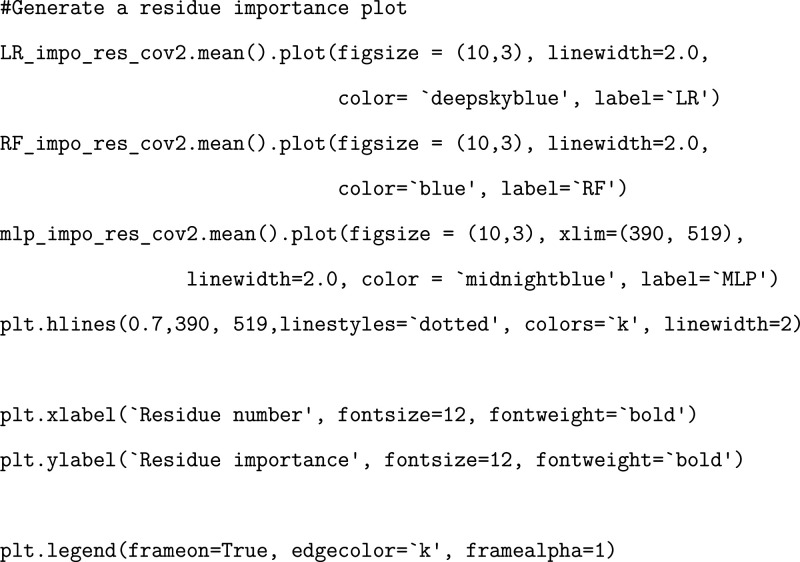


**Figure 5:**
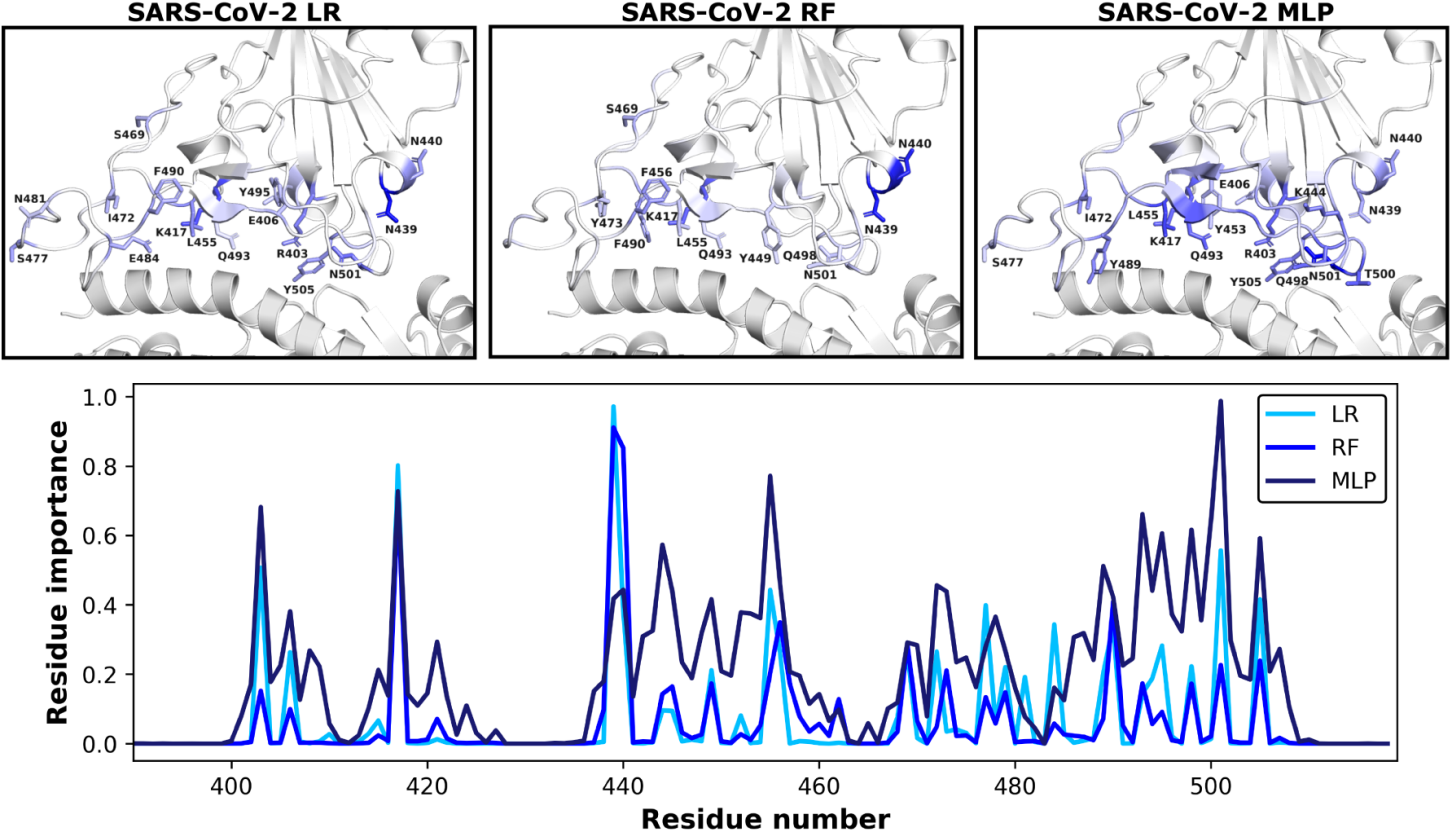
Visualization of calculated residue importance via three different ML models as described in this tutorial utilizing our truncated data set. Importances are plotted and projected onto the SARS-CoV-2 structure; residues colored darker blue correspond to having larger calculated importances.

Any set of nonidentical ML methods (RF/LR/MLP) will not produce identical results due to the varying mathematical construction of the models. Because of this, we examine the areas of greatest overlap and individually high performance to validate the success of our approach. We note that although this tutorial uses a truncated data set, our method has successfully pinpointed important residues consistent with other studies. For instance, Chen et al. have previously used ML methods and determined that mutations at the N439 (identified by RF/LR/MLP methods), T500 (identified by MLP), N501 (identified by RF/LR/MLP), and Y505 (identified by LR/MLP) positions are associated with large changes in binding free energies between the RBD and ACE2 receptor and, thus, potentially differences in infectivity.^13^ Additionally, da Costa et al. previously discussed that notable mutations at positions N440 (identified by RF/LR/MLP), Q493 (identified by RF/LR/MLP), and Q498 (identified by RF/MLP) are associated with greater infectivity via strengthening binding affinity.^46^ We further note that regardless of ML method used, the K417, N439, and N501 positions are deemed to be important for differentiating binding (Figure 5). In particular, mutation of N501 was initially associated with the alpha variant^47^ and mutation of K417 was associated with the beta variant of SARS-CoV-2.^48,49^ Cheng et al. note that the N501Y mutation increases affinity to the ACE2 receptor via increased amino-aromatic and aromatic–aromatic interactions, and the K417N/T mutations decrease affinity, marking these two residue positions as essential in regulating the global dynamics of the RBD-ACE2 interactions.^49^ Additionally, Geng et al. have elucidated the role of the K417 residue in modifying the structural conformation of the SARS-CoV-2 RBD.^50^ Thomson et al. also found the N439 position to play a major role in determining the binding affinity of the RBD to ACE2, pointing out that the prevalent N439K mutation increases binding affinity for the ACE2 receptor.^51^ In other words, numerous studies have confirmed the impact of the highly important residues identified by this study, validating the implementation of our ML-based classification methods.

In cases where significant overlap of importances between models is not observed, we recommend investigating the individual model accuracies to guarantee they are performing within acceptable margins. The model accuracies can be improved by increasing the scope and reliability of training data (for instance by increasing the MD trajectory resolution).^23^ Further improvements can be made by tuning the hyper parameters for each model (*RF*_*tuned*_*params*/*LR*_*tuned*_*params*/*MLP*_*tuned*_*params* in this tutorial). General methods such as grid searching, random searching, and Bayesian optimization can be used to identify which parameters produce higher model accuracies.^52,53^ If the computational resources can be spared to automate parameter searches, it is recommended as optimized parameters will yield the most consistent results.^52^

## Conclusions

This tutorial demonstrates how to apply ML models to MD simulation data. We identified the key differences in the RBDs of SARS-CoV and SARS-CoV-2 that influence binding affinity. We note that this same method could be utilized to determine differences in binding interactions among SARS-CoV-2 variants, which could have implications for potential therapeutic strategies targeting the spike protein.^54^ However, comparing highly similar variants (such as the two-residue mutations T22N and F59S between KP.3 and XEC)^55^ will only yield meaningful residue importances if these mutations affect the RBD-ACE2 binding affinity. The developed technique is not limited to exploration of SARS-CoV and SARS-CoV-2 and can be applied generally to protein-receptor binding sites, such as FAB-epitope binding, as long as the binding region is well characterized.^56,57^ Our implementation of logistic regression, random forest, and MLP models provides a brief survey of different ML methods, which can be generalized for a variety of classification problems. This article serves as a practical guide, which can be utilized to effectively conduct an ML-based analysis to further biological understanding.

## Supplementary Material



## Notes

The full unabridged python analysis can be found alongside this publication as a Jupyter Notebook (ZIP)
